# Developmental brain programmes and biomarker activation in Alzheimer’s disease

**DOI:** 10.1093/braincomms/fcag337

**Published:** 2026-09-03

**Authors:** Lorenzo Pini, Monica Margoni, Massimo Filippi, Bruno Pietro Imbimbo

**Affiliations:** Department of Neuroscience, University of Padova, Padova 35122, Italy; Padova Neuroscience Center, University of Padova, Padova 35122, Italy; Veneto Institute of Molecular Medicine (VIMM), Padova 35122, Italy; Neurology Unit, IRCCS San Raffaele Scientific Institute, Milan 20132, Italy; Neuroimaging Research Unit, Division of Neuroscience, IRCCS San Raffaele Scientific Institute, Milan 20132, Italy; Neuroimaging Research Unit, Division of Neuroscience, IRCCS San Raffaele Scientific Institute, Milan 20132, Italy; Center for Alzheimer’s and Related Diseases (CARD), Neurology Unit, IRCCS San Raffaele Scientific Institute, Milan 20132, Italy; Vita-Salute San Raffaele University, Milan 20132, Italy; Neurorehabilitation Unit, IRCCS San Raffaele Scientific Institute, Milan 20132, Italy; Neurotech Hub, Vita-Salute San Raffaele University, Milan 20132, Italy; Neurophysiology Service, IRCCS San Raffaele Scientific Institute, Milan 20132, Italy; Department of Research and Development, Chiesi Farmaceutici, Parma 43122, Italy

**Keywords:** GFAP, p-tau217, sTREM2, neurodevelopment, biomarker biology

## Abstract

Recent studies show that serum glial fibrillary acidic protein (GFAP) is highest in healthy infancy and early childhood and then declines steadily with age, while plasma tau protein phosphorylated at position 217 is strikingly elevated in healthy newborns and can exceed values observed in Alzheimer’s disease (AD). Additional evidence indicates that soluble phosphorylated tau forms are not specific to AD, as increased tau protein phosphorylated-217 has been reported in *MAPT R406W* carriers without amyloid pathology and in Niemann-Pick disease type C, where plasma tau protein phosphorylated-217 is associated with disease progression and lysosomal enlargement. These observations invite a broader review of biomarker biology across development, physiological remodelling and neurodegeneration. We hypothesize that, at least in part, increases of GFAP, tau protein phosphorylated-217 and related biomarkers in AD reflect a chronic attempt of the ageing brain to reactivate multicellular programmes that are physiologically engaged during development, repair and circuit refinement. In this framework, biomarker elevation does not necessarily indicate that the reactive programme is intrinsically toxic. Rather, persistent elevation may index the duration and intensity of unresolved metabolic, inflammatory, vascular, proteostatic, mitochondrial or infectious stressors that keep repair and homeostatic systems engaged until their compensatory capacity is exceeded. Protein precipitation is therefore positioned as a downstream consequence of persistent stress, chronic compensatory activation, increased protein turnover, secretion or processing and impaired clearance or proteostasis. This *Review* summarizes the developmental biomarker evidence, examines alternative interpretations and discusses therapeutic implications focused on upstream stressors and failed resolution rather than biomarker lowering alone.

## Graphical Abstract

**Graphical Abstract:**
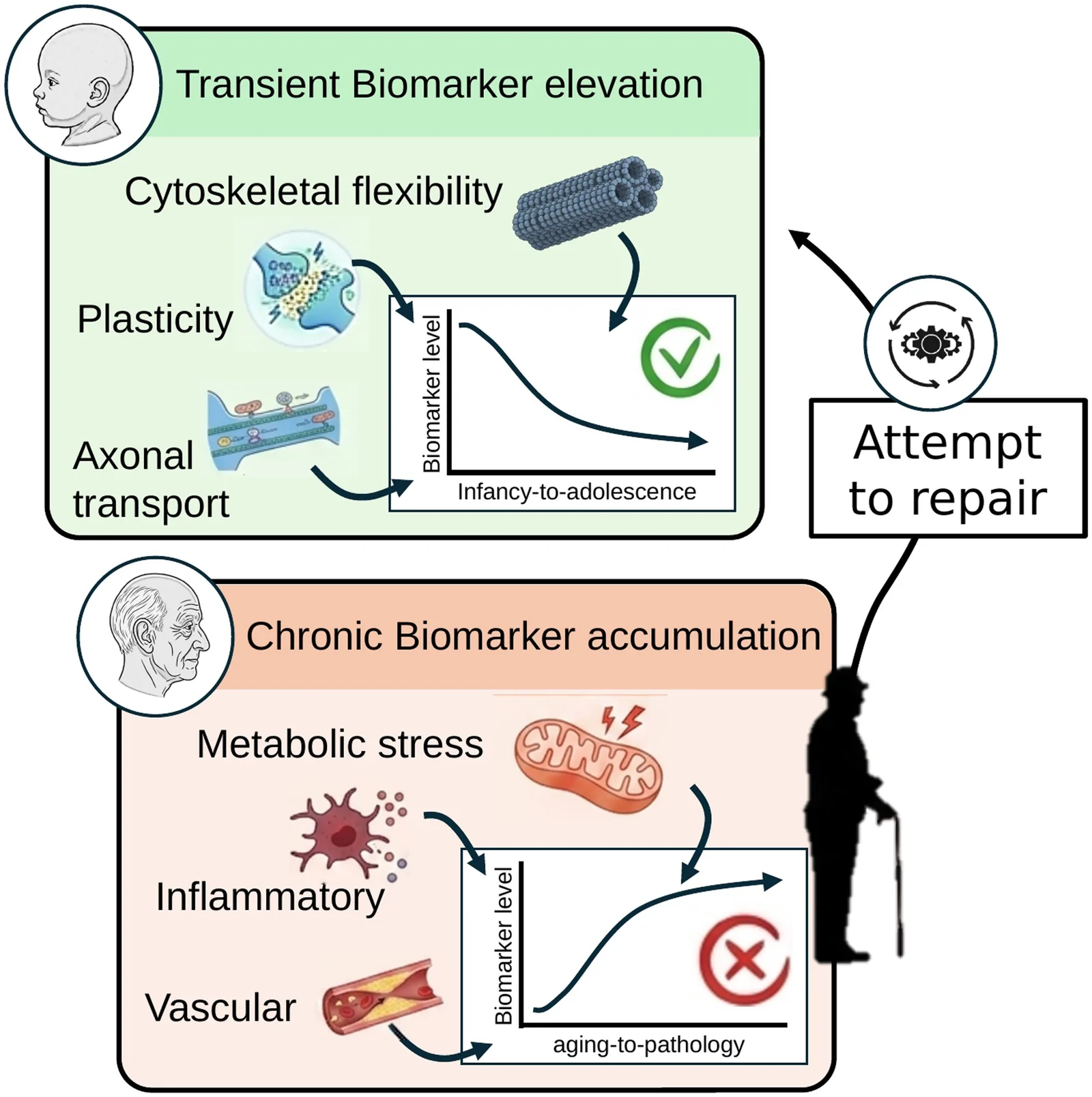

## Teaser

Pini *et al*. review developmental biomarker changes and propose that elevations of phosphorylated-tau-217 and glial fibrillary acidic protein in Alzheimer'’s disease may reflect unresolved adaptive brain programmes rather than intrinsically toxic responses. The review emphasizes upstream stressors, failed resolution and therapeutic strategies restoring homeostasis.

## Introduction

**Figure 1:**
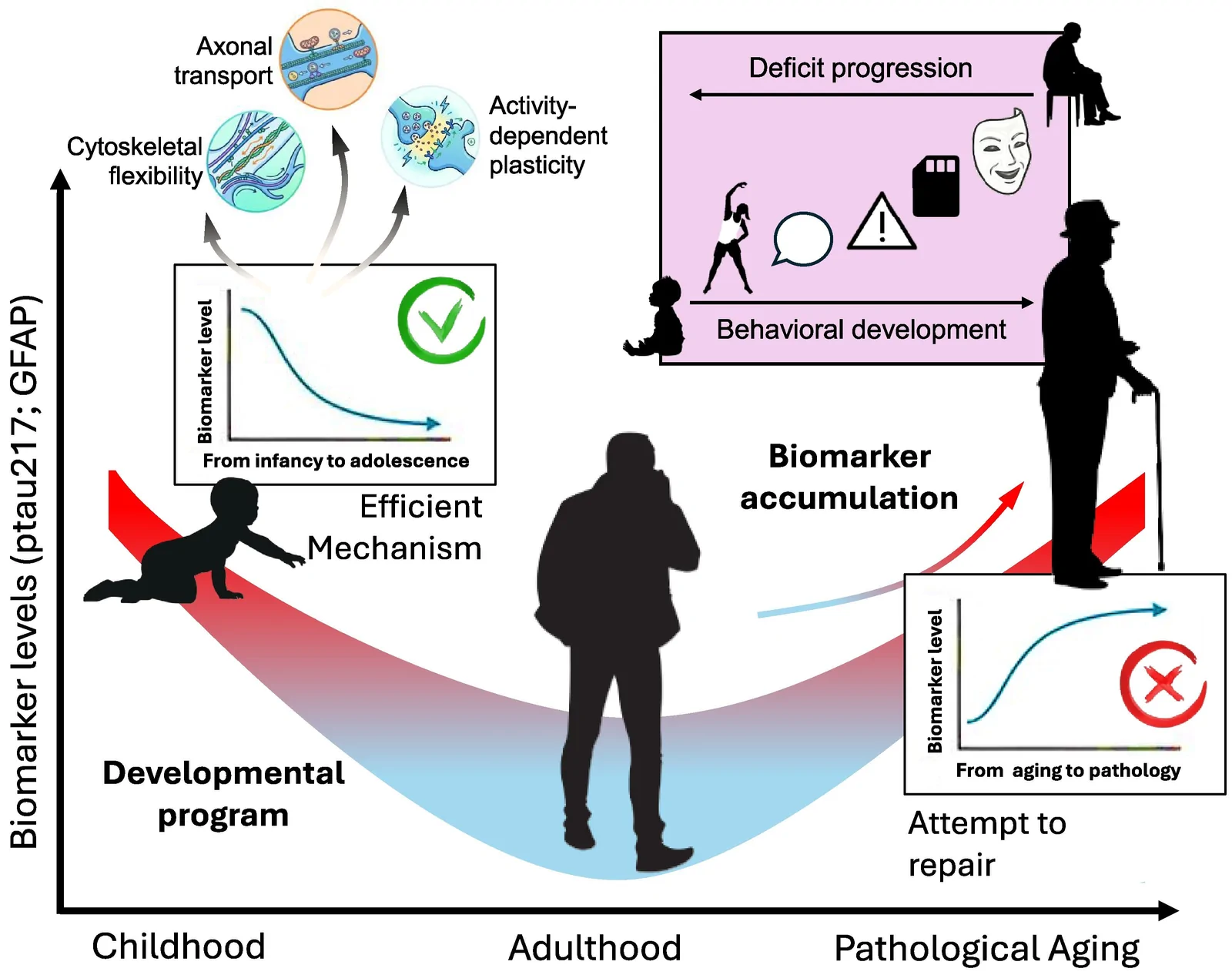
**Lifespan model of biomarker dynamics and failed resolution in Alzheimer’s disease.** During early brain development, elevated biomarkers (e.g. p-tau217 and glial fibrillary acidic protein) can reflect coordinated and efficient multicellular programmes supporting circuit maturation, followed by physiological decline. In Alzheimer’s disease, persistent metabolic, inflammatory, vascular, proteostatic, mitochondrial or infectious stressors may sustain repair and homeostatic responses that remain insufficient to restore tissue integrity. Protein precipitation is therefore positioned as a downstream consequence of persistent stress, chronic compensatory activation, increased protein turnover or processing and impaired clearance or proteostasis. Boxes illustrate the contrast between transient developmental signalling and persistent late-life biomarker accumulation, alongside the retrogenesis principle linking behavioural decline to reversed developmental trajectories.

Alzheimer’s disease (AD) has long been interpreted within a protein-centred framework in which abnormal increases in soluble and insoluble biomarkers are considered primary drivers or indicators of neurodegeneration. The growing dissociation between biomarker modulation and clinical efficacy suggests that this picture may be incomplete, as shown by several tau-directed therapeutic programmes. In the phase-2 TANGO study, gosuranemab (humanized IgG4 monoclonal anti-tau antibody) produced robust target engagement, with marked reductions in cerebrospinal fluid (CSF) unbound N-terminal tau and modest reductions in CSF p-tau181, yet no beneficial clinical effects were observed, and no effect was detected on tau PET progression.^1^ More recently, the phase-2b Auτonomy study of posdinemab (anti-p-tau217 monoclonal antibody) was discontinued following an interim review showing that, despite strong suppression of CSF p-tau217, the treatment did not achieve statistical significance in slowing clinical decline in early AD [2026 AD/Parkinson’s disease (PD) conference]. These negative trials are notable not only because they challenge the validity of therapeutic target but also because they highlight an increasing dissociation between biomarker modulation and clinically meaningful outcomes. These findings raise a fundamental biological question. If the reduction of soluble p-tau species is not sufficient to alter disease progression, could at least part of the biomarker increase in AD reflect an endogenous but ineffective attempt of the brain to resist injury rather than a purely toxic process? This question is reinforced by growing evidence that tau phosphorylation is not inherently pathogenic but plays important physiological roles in cytoskeletal flexibility, axonal transport and activity-dependent plasticity across the lifespan, while also lacking a clear-cut distinction between normal and pathological states, as tau modification patterns are highly similar and variable across healthy and diseased conditions.^2^ Importantly, the increase in soluble p-tau species is not restricted to AD. Sato *et al*.^3^ showed that *MAPT R406W* carriers can have increased CSF T217 phosphorylation occupancy despite a normal amyloid-β 42/40 ratio, and Gonzalez-Ortiz *et al*.^4^ reported increased plasma p-tau217 in Niemann-Pick disease type C, where levels correlated with progression and lysosomal enlargement. These findings support the view that p-tau217 can also index downstream tau biology or cellular stress outside the amyloid-positive AD continuum. Increases in soluble p-tau species are also consistent with the proteinopenia framework in which aggregated proteins may represent common downstream denominators of heterogeneous biological, toxic or infectious drivers rather than universal primary causes.^5,6^ Building on recent observations that serum glial fibrillary acidic protein (GFAP) and plasma p-tau217 are markedly elevated during normal early brain development, in some cases exceeding levels observed in AD, this Review summarizes the available evidence that multiple cell populations, including neurons, astrocytes, microglia, oligodendrocytes and neural stem cells, may enter a chronic stress-response state in the ageing AD brain. We review whether progressive increases in GFAP, soluble triggering receptor expressed on myeloid cells-2 (sTREM2), p-tau217 and related proteins may represent an adaptive but ultimately insufficient reparative programme that resembles developmental brain activation and may culminate, when upstream stressors persist, in abnormal protein precipitation and aggregation (Fig. 1). To this aim, first we reviewed developmental biomarker data, then we examined retrogenesis and network maturation concepts, discussing glial, neuronal, oligodendroglial and stem-cell responses in AD. In the final sections we considered alternative interpretations and therapeutic implications.

## Developmental biomarker biology and its relevance to AD

Before examining developmental biomarker biology, it is useful to situate the present hypothesis within Reisberg’s retrogenesis hypothesis (RH), which posits that the order of functional decline in AD is the inverse of normal development. Abilities acquired last during maturation are lost first, while those established earliest are lost last.^7^ Sensorimotor cortices are among the earliest regions to reach myelination maturity, whereas structural maturation continues into adulthood in associative-polymodal regions.^8^ AD preferentially affects brain regions that mature later, such as the hippocampus and association cortices. Further, the final stage of the Global Deterioration Scale resembles the functional profile of newborns, including speech limited to a few words, loss of motor abilities, stupor and coma. Similar ontogenic principles have been observed in other neurodegenerative conditions.^9^ The RH therefore provides a rationale for expecting that cellular programmes active during brain maturation may be re-engaged during neurodegeneration. This framework has direct bedside relevance, because it predicts that clinical decline should not be interpreted simply as passive loss, but as the progressive failure of higher-order systems that are last to mature and first to lose compensatory reserve.

An illustration of this last-in-first-out principle at the motor cortex level has recently emerged from functional neuroimaging. Gordon *et al*.^10^ demonstrated that the motor homunculus is not a continuous somatotopic map but is interrupted by an integrative system, the somato-cognitive action network (SCAN), a set of inter-effector regions alternating with classical foot, hand and mouth representations along the precentral gyrus, interconnected with cortical regions responsible for action planning, physiological regulation, arousal and autonomic control. The neonatal motor cortex shows effector-specific connectivity but lacks this integrative inter-effector architecture. By 11 months of age, the SCAN becomes identifiable and is nearly adult-like by age 9. In PD, functions mediated by SCAN-linked structures deteriorate, with consequences for postural instability, autonomic dysfunction and reduced self-initiated activity, symptoms that mirror the integrative coordination functions of the SCAN.^11^ This provides a system-level embodiment of the RH in motor cortex. The SCAN is absent at birth, built through postnatal developmental programmes, essential for integrated action in the mature brain and selectively vulnerable to neurodegeneration. This developmental arc parallels biomarker changes and supports the hypothesis that cellular programmes recruited during brain development may be reactivated by the ageing and diseased brain.

The idea that neurodevelopmental and neurodegenerative processes share molecular pathways is not new. Mattsson *et al*.^12^ reported that CSF p-tau181 levels are markedly elevated in human neonates, with most preterm infants exceeding the upper quantification limit of the assay. Similarly, CSF total tau, alpha-sAPP and beta-sAPP show high concentrations early in life, followed by a progressive decline during the first years. These findings support the view that elevated tau phosphorylation is part of a physiological developmental programme, likely associated with increased neuronal plasticity and structural remodelling. Convergent molecular pathways may be aberrantly re-engaged in AD, raising the possibility that tau phosphorylation may reflect a response to synaptic stress rather than a purely pathological event. Similar mechanisms have been suggested for p-tau217 in newborns and AD.^13^

Two recent evidence lines are equally provocative regarding GFAP. In healthy children, serum GFAP is highest in early life and then falls across childhood and adolescence. Tybirk *et al*.^14^ showed a steep age-related decline from infancy to adolescence, while Stukas *et al*.^15^ independently confirmed high early-life GFAP and a continuous fall thereafter. Similarly, serum NfL, a marker of axonal damage, displays highest and most variable levels in young children, decreasing by up to 10% per year in the first 5 years and stabilizing during adolescence. Serum total tau displayed a similar decline, with the greatest rate of change before age four.^15^ These data suggest that axonal remodelling represents a hallmark of brain development and that NfL, often viewed purely as a neurodegeneration marker, may also report physiological structural turnover. In parallel, Gonzalez-Ortiz *et al*.^13^ reported that plasma p-tau217 in healthy newborns is not only higher than in older healthy groups but can even exceed levels measured in biologically defined AD, with premature infants showing a decline of serum p-tau217 over the first months of life. Together with evidence linking p-tau217 to core AD mechanisms in adults,^16,17^ these results indicate that substantial increases in canonical AD biomarkers can also occur in physiological conditions characterized by structural remodelling, rather than exclusively reflecting irreversible degeneration.

The newborn brain shows that high p-tau217 can be coupled to plasticity rather than insoluble tau aggregation. Phosphorylation modulates tau affinity for microtubules, increasing cytoskeletal flexibility required for axonal and synaptic growth.^2^ Astrocytes also undergo major maturation early in life. High circulating GFAP in infancy aligns with intense astrocytic remodelling and astrocyte-neuron metabolic coupling during rapid synaptogenesis.^14,15^ Astrocytes are active participants in brain repair, as GFAP-expressing cells in the adult brain retain neural stem cell properties. Butruille *et al*.^18^ demonstrated in the hypothalamus of experimental models that GFAP-positive cells form a population of neural stem/progenitor cells capable of generating neurospheres in vitro and differentiating into neurons, astrocytes and oligodendrocytes. Approximately 80% of these GFAP-positive cells co-expressed the stem cell marker Sox2, confirming their multipotent identity and suggesting that GFAP elevation can reflect active cellular renewal rather than terminal injury. Additional support comes from Sachewsky *et al*.^19^, who identified a rare population of primitive neural stem cells in the adult mammalian periventricular region that are GFAP-negative, responsive to leukaemia inhibitory factor and express the pluripotency marker Oct4. These adult-derived primitive neural stem cells generate GFAP-positive definitive neural stem cells both in vitro and in vivo. After selective ablation of all GFAP-expressing neural stem cells, primitive neural stem cells repopulated the depleted stem-cell compartment over time. After experimental stroke, this population expanded 5.4-fold within four days, preceding expansion of GFAP-positive neural stem cells. These findings support a hierarchical model in which primitive stem-cell reserves can be activated by injury signals. Microglia participate in physiological synaptic pruning and circuit sculpting. Oligodendrocyte precursor cells proliferate and differentiate to support rapid myelination, protecting axons and improving conduction across development.^20^ Neural stem and progenitor cells contribute to postnatal neurogenesis and circuit expansion. Importantly, these cell types do not act independently but form an integrated developmental consortium. A rise in biomarkers during this phase is therefore expected, as cellular turnover, structural remodelling and protein trafficking are accelerated. Thus, biomarkers such as GFAP and p-tau217 should not automatically be interpreted as simple readouts of cell damage or death. In some settings, such as infancy, they may instead report active cellular work. The same likely applies to sTREM2, which reflects TREM2 shedding from activated microglia and is generally interpreted as a marker of microglial response rather than a direct marker of tissue necrosis.^21,22^

This developmental perspective is reinforced by Brum’s hibernation study, building on earlier work showing that metabolic rate depression and tau phosphorylation are physiologically linked during mammalian hibernation.^23^ In bears and hamsters, reversible physiological tau hyperphosphorylation occurred during torpor.^24^ Relevant phospho-sites overlapped with those emphasized in AD, including p-tau217, yet there was no corresponding increase in microtubule-binding region fragments that signal tangle-associated aggregation. The lesson is that the biological meaning of biomarker elevation depends on context, reversibility and the success or failure of the surrounding cellular response. Here we present the idea that AD partly recapitulates a broad developmental and repair-like programme that normally helps the brain build, prune and stabilize circuits. In the child, the programme is efficient and culminates in maturation. In AD, the same families of responses are repeatedly recruited but cannot resolve the underlying stressors. Biomarker levels therefore rise because the brain remains locked in an attempt at repair that no longer restores homeostasis. Key findings from these studies are summarized in Table 1.

**Table 1:** Key findings from the literature that support the proposed hypothesis

Study	Material	Core finding	Contribution to the present hypothesis
Abdelhak *et al*. 2022^25^	Review of blood GFAP across neurological disorders	GFAP reflects astrocytic injury and activation in multiple CNS conditions.	Supports use of GFAP as a dynamic astrocytic response marker rather than a simple end-stage marker.
Tybirk *et al*., 2023^14^	391 children aged 0.4 to 17.9 years	Serum GFAP is highest and most variable in infants and declines steeply with age.	Shows that markedly elevated GFAP can be physiological in early life.
Stukas *et al*., 2024^15^	CALIPER cohort of 316 healthy children	GFAP is highest at age 1 to <3.5 years and falls continuously through adolescence.	Independent replication of age-linked developmental GFAP biology.
Sato *et al*., 2021^3^	CSF in AD and non-AD tauopathies	*MAPT R406W* carriers showed increased CSF T217 phosphorylation occupancy with normal amyloid-beta 42/40 ratio.	Shows that p-tau217 is not specific to AD and can reflect downstream tau biology.
Gonzalez-Ortiz *et al*., 2024^4^	Plasma in Niemann-Pick disease type C and AD	Plasma p-tau217 was increased in Niemann-Pick disease type C and associated with disease progression and lysosomal enlargement.	Supports the interpretation of p-tau217 as a marker of broader tau and lysosomal stress outside AD.
Gonzalez-Ortiz *et al*., 2025^13^	462 participants across newborns, controls and AD	Plasma p-tau217 is higher in healthy newborns than in adults and even higher than in AD.	Shows that strong p-tau217 elevation can occur in non-degenerative physiology.
Suárez-Calvet *et al*., 2016^21,22^	CSF in early symptomatic and dominantly inherited AD	sTREM2 rises after amyloid and neuronal injury changes.	Consistent with microglial activation as a secondary adaptive response.
Espay, 2023^5^	Conceptual review of precision medicine in neurodegeneration	Aggregated pathology may be a common downstream denominator of heterogeneous biological drivers.	Supports a framework focused on upstream drivers rather than universal protein toxicity.
Ezzat *et al*., 2023^6^	Proteinopenia paradigm review	Aggregation and depletion of soluble functional protein pools can occur simultaneously.	Supports therapeutic consideration of soluble monomer restoration and proteostasis.
Hong *et al*., 2016^26^	AD experimental models	Complement and microglia mediate early synapse loss before overt plaques.	Suggests pruning is an early active process that may begin as attempted circuit stabilization.
Sachewsky *et al*., 2014^19^	Adult mammalian periventricular neural stem-cell niche	Primitive neural stem cells generate GFAP-positive definitive neural stem cells and expand after injury.	Provides mechanistic support for an injury-responsive stem-cell reserve upstream of GFAP-positive cells.
Moreno-Jiménez *et al*., 2019^27^	Human post-mortem hippocampus	Adult hippocampal neurogenesis is abundant in healthy brains and sharply reduced in AD.	Supports the idea that neurogenic compensation exists but fails in AD.
Zhou *et al*., 2020^28^	Single-nucleus transcriptomics	Reactive microglial and oligodendrocyte states are present in AD.	Supports a multicellular disease programme that includes myelin-related adaptation.
Brum *et al*., 2025^24^	Hibernating bears and hamsters plus AD brain tissue	Reversible p-tau elevations occur without MTBR tau aggregation in hibernation.	Separates adaptive phosphorylation from irreversible tau aggregation.

## Activation of developmental and repair-like programmes in AD

AD can be re-read through this developmental lens. Neurons exposed to amyloid stress, metabolic insufficiency, oxidative injury and synaptic dysfunction may attempt to remodel their cytoskeleton and preserve transport by re-engaging phosphorylation programmes useful earlier in life. This possibility finds indirect support in a two-photon longitudinal imaging study by Zwang *et al*.^29^, who demonstrated in AD experimental models that neurons harbouring neurofibrillary tangles died more than three times less frequently than tangle-free neurons, leading the authors to hypothesize that tangle formation may represent a cellular senescence mechanism that protects neurons from apoptosis. This observation does not prove that tangles are protective in humans, but it cautions against assuming that every protein-positive state is the primary driver of cell death. Astrocytes become reactive and increase GFAP expression. This response is initially plausible as a supportive manoeuvre because astrocytes buffer ions, recycle neurotransmitters, provide metabolic substrates and contribute to barrier and vascular regulation.^30^ GFAP has therefore emerged as a broad marker of astrocytic activation across neurological disorders, not solely as an end-stage marker of tissue destruction.^25^ Microglia are likewise recruited to sense damage, remove debris and prune dysfunctional synapses. CSF sTREM2 rises in the early symptomatic phase of AD after amyloid-related and neuronal injury markers have already changed, consistent with a secondary but active microglial response.^21,22^

Oligodendrocytes and their precursor cells may represent another underappreciated arm of this attempted rescue. Single-nucleus transcriptomic studies have identified reactive oligodendrocyte states in AD and broader TREM2-dependent and TREM2-independent multicellular responses that include glial remodelling.^28^ Reviews of oligodendrocyte lineage biology also support the view that myelin maintenance is an active and vulnerable process across the lifespan.^20^ A reasonable inference is that the AD brain attempts to preserve axonal function through neuronal remodelling, reprogramming of glial support and myelin maintenance. This remains an inference from available transcriptomic and developmental data rather than proof that oligodendroglial responses are sufficient to rescue cognition.

Neural stem cells could add a final compensatory layer. Human data suggest that adult hippocampal neurogenesis declines sharply in AD,^27,31^ and this has motivated the view that AD may partly be understood as a disorder of impaired neurogenesis and failed cellular renewal,^32^ as observed in other neurological conditions.^33^ AD may therefore be understood not only as neuronal loss but also as failed cellular replacement and failed maintenance of plasticity niches. We therefore hypothesize that AD recruits several components of the multicellular programme used during development, but in an ageing molecular environment that lacks the capacity to terminate the response successfully. This hypothesis remains compatible with alternative models in which the same biomarker elevations reflect cell injury, altered clearance or upstream metabolic and inflammatory stress rather than developmental activation per se.

A further implication of this framework concerns individuals who exhibit elevated biomarker levels yet maintain preserved cognitive function over extended periods. Such cases, often described within the spectrum of preclinical AD or resilience phenotypes, may reflect a condition in which the same multicellular adaptive programme is successfully engaged and effectively regulated. In these individuals, increased levels of p-tau217, GFAP or sTREM2 may signal an active but still efficient compensatory response capable of stabilizing network function despite ongoing stressors. Rather than indicating inevitable progression, biomarker elevation in this context may correspond to a dynamic mechanism sufficiently coordinated to preserve functional integrity. Thus, the critical transition towards clinical decline may not be determined solely by the magnitude of biomarker change, but by the progressive loss of the system’s ability to resolve and terminate the adaptive response.

## From persistent stress to failed resolution and biomarker accumulation

AD may not be a failure to respond, but a failure to resolve. Such a model shifts attention from static to dynamic disease biology. Patients may be better stratified by whether biomarker trajectories indicate a contained adaptive response or a persistent state associated with impending functional decline. In this view, the central issue is not the activation of cellular and molecular programmes aimed at preserving brain integrity, but the progressive inability of these responses to reach a stable and self-limiting endpoint. When neurons, astrocytes, microglia and oligodendroglial lineages are repeatedly activated, the release, shedding or leakage of cell-associated proteins into interstitial fluid, CSF and blood will rise. GFAP increases as astrocytes become structurally reactive.^25^ sTREM2 increases as microglial TREM2 is cleaved during activation.^21^ P-tau217 increases as neurons engage cytoskeletal remodelling pathways, and in AD this signal tracks closely with established amyloid and tau biomarker change.^16,17^ Aβ42 may also reflect altered production, mobilization or trafficking during attempts to stabilize synapses, in line with biomarker trajectory studies showing coordinated shifts in amyloid-related and downstream injury markers over the course of disease.^34^

The crucial divergence from development lies in efficiency and resolution. In children, high biomarker production is time-limited and embedded in successful circuit construction. Clearance mechanisms, proteostasis, lysosomal processing and vascular drainage are adequate for the task. In AD, the same type of response is superimposed on ageing, chronic inflammation, impaired proteostasis, mitochondrial dysfunction, oxidative stress and reduced clearance, creating a state in which the initiating stressors could remain unresolved over years.^35^ Persistent biomarker elevation should therefore be interpreted primarily as evidence of unresolved biological stress and exceeded compensatory capacity, rather than as direct proof that the repair response has become toxic. One plausible sequence is persistent stress, chronic compensatory or reactive activation, increased protein turnover, secretion or processing, impaired clearance or proteostasis and eventual precipitation or sequestration. This sequence is consistent with longitudinal models in which amyloid accumulation precedes phospho-tau and glial biomarker progression.^34^ It places protein precipitation downstream of persistent stress and failed resolution. It does not claim that soluble p-tau217, GFAP or sTREM2 alone cause AD. In other words, failure is not equivalent to causation.

Pruning is central to this framework. In development, it removes weak or unnecessary synapses and improves network efficiency. The mechanisms governing this process are well characterized: activity-dependent synaptic pruning in the developing mammalian CNS relies on coordinated signals among neurons, microglia and astrocytes, with complement-mediated tagging and phagocytic elimination of less active synapses.^36^ In AD, the same complement-microglial machinery may be re-engaged aberrantly.^26^ This is often described purely as pathology. Yet a more nuanced interpretation is possible. Pruning may begin as an attempt to eliminate failing synapses and protect network stability. The problem is that in AD the threshold for successful pruning, replacement and rewiring is not met. Synapses may be removed faster than healthy circuits can be rebuilt. In this sense, the pathogenic burden may reside in persistent upstream stress and failed reconstruction rather than in the existence of pruning as a biological process.

The same logic applies to tau phosphorylation. As noted above, tau phosphorylation can be a reversible physiological response during hibernation.^23,24^ In AD, the analogous response appears to lose reversibility. Short-lived adaptive phosphorylation may be replaced by chronic phosphorylation, impaired dephosphorylation, altered trafficking, reduced clearance and eventual insoluble aggregation. This concept of failed reversibility may unify several biomarkers. Rising GFAP, sTREM2 and p-tau217 can each be read as evidence that the brain is still responding. What makes AD devastating is not absence of response, but the inability of the response to restore a stable endpoint. A protective mechanism that ultimately fails should not automatically be reclassified as the primary disease driver. Cardiac hypertrophy, insulin secretion, neuroinflammation, gliosis and synaptic remodelling are examples of compensatory responses that may become insufficient when the driver persists, without necessarily constituting the initiating cause of disease.

## Alternative interpretations and limits

Several caveats follow from this synthesis. First, developmental parallels do not prove developmental identity. Similar biomarkers may arise from distinct mechanisms in newborns, hibernation, Niemann-Pick disease type C and AD. Second, plasma and CSF concentrations integrate production, secretion, leakage, clearance and assay-specific effects, so they cannot by themselves identify the cellular source or causal direction of a process. Third, evidence for oligodendroglial, microglial and neural stem-cell participation in AD remains partly indirect and should be tested with longitudinal multimodal studies that combine biofluids, PET, MRI, single-cell data and clinical trajectories. Finally, the model does not deny that aggregated proteins can contribute to downstream injury. It instead distinguishes contribution from primacy and proposes that biomarker elevation should be interpreted within the broader context of unresolved upstream stressors and failed homeostatic resolution.

## Therapeutic implications: targeting upstream stressors and failed resolution

These studies invite a shift in perspective. AD may involve not only degeneration, but also chronic and insufficient activation of developmental and repair-like brain programmes. Biomarker elevation could thus reflect a brain still attempting to adapt but unable to complete the process. This interpretation does not exclude that tau dysregulation, amyloid deposition or glial activation contributes to neurotoxicity and neurodegeneration. Rather, it suggests that these processes are context-dependent, with their impact shaped by timing, reversibility, persistence of upstream drivers and the surrounding cellular environment. Therapeutically, this model does not prioritize biomarker lowering as a goal in itself. The central challenge is to identify and neutralize the upstream metabolic, inflammatory, infectious, vascular, proteostatic, oxidative or mitochondrial stressors that sustain chronic activation of these pathways, while restoring the soluble monomeric fraction of proteins depleted during aggregation and improving clearance and proteostasis.^5,6^ This framework also invites closer study of oligodendrocyte biology and adult neurogenic niches in AD, areas that remain less integrated into mainstream biomarker models than astrocytes, microglia and neurons. Successful disease-modifying therapies may need to combine removal or neutralization of injurious deposits with restoration of the conditions that permit adaptive biomarker elevations to resolve without progression to disability.

## Data Availability

Data sharing is not applicable to this article as no new data were created or analysed in this study.
